# Prevalence, risk factors, and characterisation of extended-spectrum β-lactamase -producing Enterobacterales (ESBL-E) in horses entering an equine hospital and description of longitudinal excretion

**DOI:** 10.1186/s12917-024-04260-z

**Published:** 2024-09-13

**Authors:** Katarina Eskola, Elina Aimo-Koivisto, Annamari Heikinheimo, Anna Mykkänen, Tiina Hautajärvi, Thomas Grönthal

**Affiliations:** 1 Department of Equine and Small Animal Medicine, Faculty of Veterinary Medicine, University of Helsinki, Helsinki, Finland; 2 Department of Food Hygiene and Environmental Health, Faculty of Veterinary Medicine, University of Helsinki, Helsinki, Finland; 3 Food and Feed Microbiology Unit, Finnish Food Authority, Helsinki, Finland; 4 Oy 4Pharma Ltd, Turku, Finland; 5 Animal Health Diagnostics Unit, Finnish Food Authority, Helsinki, Finland

**Keywords:** Extended-spectrum beta-lactamase, Equine, Horse, Antimicrobial resistance, *Klebsiella pneumoniae*, *Escherichia coli*, *Enterobacter cloacae*, Nosocomial infection

## Abstract

**Background:**

Extended-spectrum β-lactamase -producing Enterobacterales (ESBL-E) are important zoonotic pathogens that can cause serious clinical infections, also in horses. Preventing the spread of ESBL-E, especially in the equine hospital environment, is key to reducing the number of difficult-to-treat infections. Estimating the local prevalence of ESBL-E in horses is crucial to establish targeted infection control programs at equine hospitals. We conducted a prevalence and risk factor study in equine patients on admission to an equine teaching hospital in Finland through a rectal ESBL-E screening specimen of the horse and a questionnaire.

**Results:**

The prevalence of ESBL-E in admitted horses was 3% (5/161, 95% CI 1–7%); none of the tested factors remained statistically significant in multivariate analysis, although antimicrobial treatment within three months was borderline significant (*p* = 0.052). Extended-spectrum β-lactamase -producing *Klebsiella pneumoniae* ST6179:CTX-M-15 was detected in three horses using whole-genome sequencing, which in combination with patient records suggested nosocomial transmission. *Escherichia coli* isolates were ST1250:CTX-M-1 (*n* = 1), ST1079:CTX-M-1 (*n* = 1), and ST1245:CTX-M-14 (*n* = 1). Multiple virulence genes were detected in the ESBL-E isolates. In the ESBL-E positive horses enrolled in a one-year follow-up study, ESBL-E were unlikely to be isolated in rectal screening specimens after the initial positive specimen.

**Conclusions:**

The prevalence of ESBL-E in horses visiting a veterinary teaching hospital in Finland is low, indicating an overall low prevalence estimate in the country’s equine population. No statistically significant risk factors were identified, likely due to the low number of cases. The duration of ESBL-E carriage is likely to be very short in horses.

**Supplementary Information:**

The online version contains supplementary material available at 10.1186/s12917-024-04260-z.

## Background

Extended-spectrum β-lactamase-producing Enterobacterales (ESBL-E) are opportunistic gram-negative pathogens. These bacteria are primarily part of the intestinal microbiota in animals and humans and exhibit resistance to multiple antimicrobial substances. The most common extended-spectrum β-lactamase (ESBL)-producing species in horses are *Klebsiella pneumoniae*, *Escherichia coli*, and *Enterobacter cloacae* [1, 2]. They can cause several types of clinical infections, including neonatal septicaemia, thrombophlebitis, and incisional infections in horses [3–5]. The prevention of the development and spread of antimicrobial resistance and multi-drug resistant bacteria among horses is exceptionally important, as there are few suitable antimicrobial agents available for use in equine medicine due to the sensitive digestive system of the horse. To ensure effective treatment of serious bacterial infections, maintaining the effect of these antimicrobial agents is crucial, both in horses and in other animals and humans.

Horses have multiple roles in the modern world; they are used in numerous equine sports (involving even global travel) and in riding hobbies for people of all ages. Horses are also classified as working animals and livestock in some countries [6]. Frequent movement and dense animal populations can promote effective spread of pathogens and infectious diseases [7], including antimicrobial-resistant bacteria. Previous French and Canadian studies revealed medical treatment within three months, number of staff at farm, and recent participation at an equestrian event as risk factors for colonisation with multidrug-resistant or ESBL-producing *E. coli* in healthy horses [8, 9]. Riding schools are at a greater risk of housing ESBL- or AmpC cephalosporinase-positive horses than breeding facilities in France [9]. People working in the equine sector and hobbyists are in close contact with horses and often exposed to horse faeces, which can pose a public health concern as ESBL-E are transmitted via the faeco-oral route and can spread between humans and animals [10]. Similar ESBL-E isolates were found in a horse, a staff member, and surfaces at an equine clinic in the Czech Republic [11]. Owning a horse is a risk factor for ESBL-E colonisation in humans [12].

The present study was initiated as part of the development of the infection control program at the Equine Veterinary Teaching Hospital (EVTH) at the University of Helsinki, Finland. For the program to be effective in a specific veterinary setting, it should reflect the animal patient population and pathogen introduction risks of that setting [13]. As the EVTH receives equine patients nationwide, estimating the national prevalence of ESBL-E carriage was required, as this has not been previously studied in Finland. Such information is crucial for evaluating the risk that horses pose to both human and animal health. In France, 29% of premises housed healthy horses shedding *E. coli* non-susceptible to the third-generation cephalosporine ceftriaxone [9]; in the Netherlands the proportion of ESBL/AmpC positive horses was 11% [14]. We hypothesized the number to be lower in Finland than in these countries, since in previous literature the prevalence of ESBL *E. coli* in healthy dogs and humans in Finland was 5% [15] and 6.3% [16], respectively; we deduced that this could reflect the prevalence also in horses. The occurrence of ESBL-E in horses has not previously been studied extensively in Finland.

Little is known about the dynamics of ESBL-E shedding in horses, as the majority of the data were collected in cross-sectional studies. One three-week study on the effect of antimicrobial treatment regimens in the ESBL-E excretion in hospitalised horses was performed and a two-month investigation on the shedding of *E. coli* in hospitalised and non-hospitalised horses treated with antimicrobials [17, 18]. We designed a study covering a longer period to evaluate if the likelihood of shedding ESBL-E diminished over time. The excretion of ESBL-E has been studied in dogs and cats and the duration of carriage appears to be limited, lasting from weeks to some months [19, 20]. These studies were conducted in the Netherlands, Portugal, and the United Kingdom.

This study aimed (1) to estimate the prevalence of ESBL-E in horses entering a Finnish equine hospital, (2) to determine the species, phenotypical antimicrobial resistance, resistance and virulence genes and sequence types of the isolated ESBL-E, and (3) to describe the longitudinal shedding of ESBL-E in rectal specimens of horses in Finland.

## Results

### Prevalence and risk factor study

#### Description of the study population

**Fig. 1:**
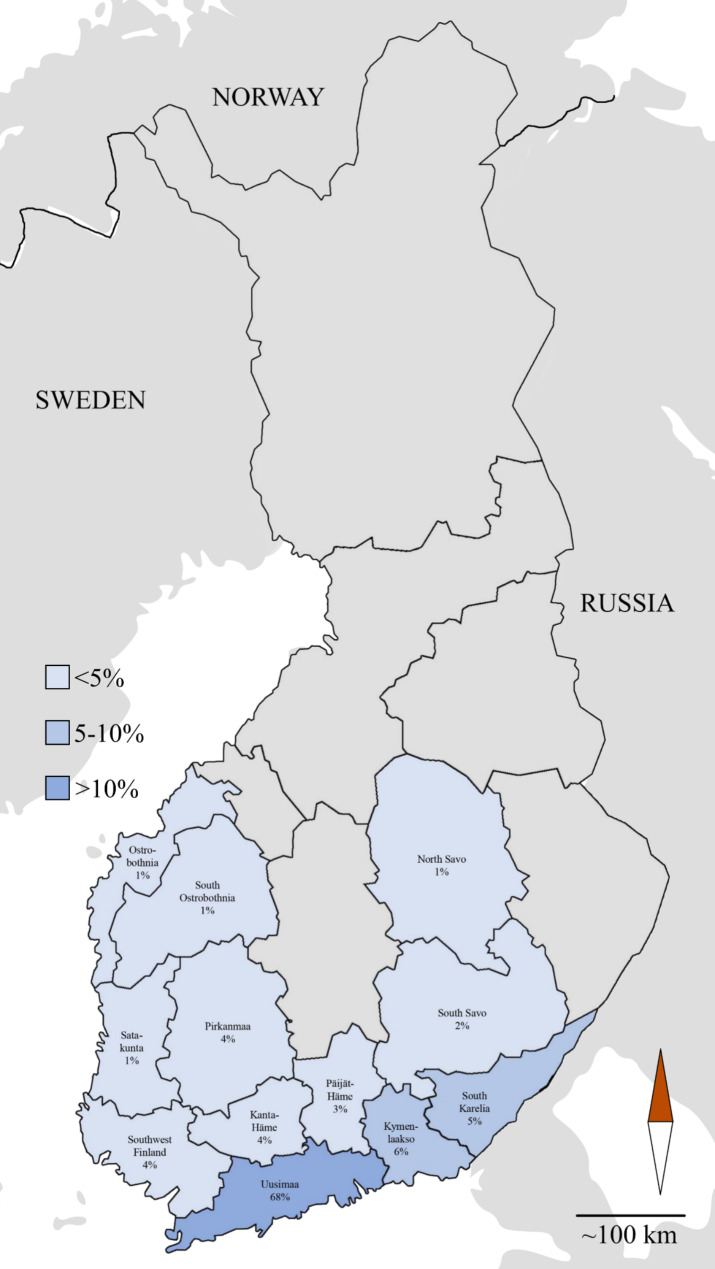
Geographical distribution of the participant horses of the ESBL-producing Enterobacterales prevalence study

Altogether 161 horses were enrolled in the study during October 2020 to April 2021. Among the sampled horses, 53% (*n* = 85) were geldings, 40% (*n* = 64) were mares, and 7% (*n* = 12) were stallions. Mean age of the horses was 10.3 years (range 0 to 30 years), and median age was 10 years. Based on the postal code of the home stable, most of the horses were from the Uusimaa region (68%), which also hosts the EVTH in the metropolitan area (Fig. 1). Most were outpatients (95%), as only eight horses (5%) were admitted to daytime emergency service. The most common presenting complaints were lameness (*n* = 22), eye examination (*n* = 20), shoeing (*n* = 15), suspected or confirmed sand ingestion (*n* = 14) and planned surgery (*n* = 12). Close to half of the study population were Warmbloods (44%, *n* = 71), 16% (*n* = 25) were Finnhorses, 14% (*n* = 23) ponies, 10% (*n* = 16) Standardbreds, and 16% (*n* = 26) other breeds.

#### Estimated prevalence of ESBL-E

Out of the 161 horses admitted to the EVTH, five (3%) harboured ESBL-E (95% CI 1–7%). Four of the horses harboured one ESBL-E isolate and one horse (horse no. 2) two ESBL-E isolates (Table 1).

**Table 1:** Phenotypical expression of antimicrobial resistance in disc diffusion test by the ESBL-producing Enterobacterales isolates

Horse ID	Isolate ID	ENA/Institut Pasteur accession no.	Isolation date (dd.mm.yyyy)	Species	Phenotypical resistance													
					Ampicillin	Amoxicillin-clavulanic acid	Cefpodoxime	Cefotaxime	Ceftazidime	Meropenem	Sulfamethoxazole-trimethoprim	Enrofloxacin	Gentamicin	Amikacin	Chloramphenicol	Tetracycline	Doxycycline	Colistin
1	HE-3*	ERS 17589596	16.10.2020	*Escherichia coli*	R	I	R	R	R	S	S	I	R	S	S	R	R	S
2	HE-4*	ERS 17592100	11.01.2021	*Escherichia coli*	R	I	R	R	R	S	R	S	R	S	I	S	S	S
3	HE-5*	ERS 17592101	13.01.2021	*Escherichia coli*	R	S	R	R	R	S	R	S	R	S	R	S	S	S
2	HE-6*	22264	11.01.2021	*Klebsiella pneumoniae*	R	I	R	R	R	S	R	R	R	S	S	R	R	S
4	HE-8*	22265	27.01.2021	*Klebsiella pneumoniae*	R	I	R	R	R	S	R	R	R	S	S	R	R	S
5	HE-15*	22266	27.01.2021	*Klebsiella pneumoniae*	R	I	R	R	R	S	R	R	R	S	S	R	R	S
2	HE-9	NA	11.02.2021	*Klebsiella pneumoniae*	R	I	R	R	R	S	R	R	R	S	S	R	R	S
6	P-2724	NA	08.02.2021	*Enterobacter cloacae*	R	R	R	R	R	S	R	R	R	S	R	R	R	S
7	P-2730	NA	15.02.2021	*Enterobacter cloacae*	R	R	R	R	R	S	R	R	R	S	R	R	R	S
7	P-2829	NA	08.05.2021	*Enterobacter cloacae*	R	R	R	R	R	S	R	I	R	I	R	R	R	S
8	P-2739	NA	19.02.2021	*Enterobacter cloacae*	R	R	R	R	R	S	R	R	R	S	R	R	R	S
9	P-2758	NA	02.03.2021	*Escherichia coli*	R	I	R	R	R	S	R	S	S	S	S	R	R	S
10	P-2771	NA	11.03.2021	*Enterobacter cloacae*	R	R	R	R	R	S	R	I	R	I	R	R	R	S
10	HE-14	NA	11.08.2021	*Escherichia coli*	R	S	R	R	S	S	R	S	R	S	R	S	S	S
10	HE-17	NA	09.09.2021	*Escherichia coli*	R	S	R	R	S	S	R	S	R	S	R	S	S	S
11	HE-10	NA	20.03.2021	*Enterobacter cloacae*	R	R	R	R	R	S	R	R	R	S	R	R	R	S
12	HE-11	NA	21.03.2021	*Enterobacter cloacae*	R	R	R	R	R	S	R	I	R	I	R	R	R	S
13	P-2788	NA	26.03.2021	*Enterobacter cloacae*	R	R	R	R	R	S	R	R	R	R	R	R	R	S

S = susceptible, I = intermediate susceptibility, R = resistant, *Horses both in the prevalence and longitudinal studies; horses without an asterisk were recruited in the longitudinal study based on ESBL-E positive specimen in the discharge screening

#### Characterisation of the ESBL-E isolates and epidemiological investigation

Out of the six phenotypically ESBL-producing isolates, three were *E. coli* and three *K. pneumoniae*; all were multi-drug resistant (resistant to at least one antimicrobial in three or more classes) (Table 1, Additional file 1) [21]. All isolates harboured several resistance genes, including multiple towards beta-lactams (CTX-M, TEM, SHV, and OXA). The virulence genes of the isolates are shown in Additional file 2.

The *E. coli* isolates were of serotypes O_unknown_:H26 (HE-3), O6:H49 (HE-4) and O166:H14 (HE-5) and of the following different sequence types: ST1250 (HE-3), ST1079 (HE-4), and ST1245 (HE-5). The isolates represented two phylogroups: B1 (HE-3, HE-4) and E (HE-5). The plasmid replicons IncFIA(HI1) (HE-3), IncFIB(K) (HE-3), IncHI1A (HE-3), IncH1B(R27) (HE-3), IncQ1 (HE-3, HE-5), and IncI1I(Alpha) (HE-4) were identified. No shiga toxin genes were found (Additional file 2).

The *K. pneumoniae* isolates (n = 3) shared nearly identical phenotypic and genotypic antimicrobial resistance patterns (Table 1, Additional file 1) and were of a novel sequence type (ST6179). Despite harbouring *aac(6’)-lb-cr* aminoglycoside and *catB3* amphenicol resistance genes, the *K. pneumoniae* isolates did not express amikacin or chloramphenicol resistance phenotypically. The isolates HE-8 and HE-15 were indistinguishable by cgMLST, and HE-6 had three allelic differences (out of 2358 alleles) from the other isolates. Plasmid replicons IncFIB and IncFII were identified in each *K. pneumoniae* isolate.

Since the *K. pneumoniae* isolates were clonally related, patient data of the EVTH were examined to find a possible epidemiological link between the horses. The data revealed that the horses harbouring the ST6179 *K. pneumoniae* were hospitalised simultaneously at the EVTH about a month before the sampling date. Horse no. 2 (isolate HE-6) and horse no. 4 (isolate HE-8) had spent three days hospitalised at the same time, and horse no. 5 (isolate HE-15) had been hospitalised for one day at the same time with horses no. 2 and 4.

#### Risk factors for colonisation with ESBL-E

Descriptive statistics of the variables are presented in Tables 2 and 3.

**Table 2:** Descriptive statistics of continuous variables for association with harbouring ESBL-producing Enterobacterales on admission

ESBL-E	Variable	*N*	Mean (SD)	Median	SE	Minimum, Maximum	Interquartile Range
No	Number of horses at home stable	153	19.41 (15.55)	15.00	1.26	1.00, 70.00	6.00, 29.00
	Age (years)	156	10.40 (5.46)	10.00	0.44	0.00, 30.00	7.00, 13.50
Yes	Number of horses at home stable	5	25.20 (17.91)	20.00	8.01	3.00, 50.00	18.00, 35.00
	Age (years)	5	7.60 (4.45)	7.00	1.99	2.00, 13.00	5.00, 11.00
Total	Number of horses at home stable	158	19.59 (15.60)	15.50	1.24	1.00, 70.00	6.00, 30.00
	Age (years)	161	10.32 (5.44)	10.00	0.43	0.00, 30.00	7.00, 13.00

**Table 3:** Descriptive statistics of categorical variables for association with harbouring ESBL-producing Enterobacterales on admission to the Equine Veterinary Teaching Hospital

Variable	Value		ESBL-E = No (*N* = 156) *n* (%)	ESBL-E = Yes (*N* = 5) *n* (%)	Total (*N* = 161) *n* (%)
Antimicrobial treatment within three months	No		121 (77.6)	1 (20.0)	122 (75.8)
	Not known		2 (1.3)	1 (20.0)	3 (1.9)
	Yes		33 (21.2)	3 (60.0)	36 (22.4)
Previously carrier of MRSA or ESBL	No		144 (92.3)	4 (80.0)	148 (91.9)
	Not known		5 (3.2)	0 (0.0)	5 (3.1)
	Yes		7 (4.5)	1 (20.0)	8 (5.0)
Housed in other than individual stall/open shed	No		149 (95.5)	5 (100.0)	154 (95.7)
	Yes		7 (4.5)	0 (0.0)	7 (4.3)
Housed in open shed	No		134 (85.9)	5 (100.0)	139 (86.3)
	Yes		22 (14.1)	0 (0.0)	22 (13.7)
Housed in individual stall	No		28 (17.9)	0 (0.0)	28 (17.4)
	Yes		128 (82.1)	5 (100.0)	133 (82.6)
Surgical procedure within three months	No		138 (88.5)	2 (40.0)	140 (87.0)
	Yes		18 (11.5)	3 (60.0)	21 (13.0)
Regularly handled by a person working at a pig farm	No		150 (96.8)	5 (100.0)	155 (96.9)
	Not known		2 (1.3)	0 (0.0)	2 (1.3)
	Yes		3 (1.9)	0 (0.0)	3 (1.9)
	Missing		1	0	1
Regularly handled by a person working in healthcare	No		104 (67.5)	4 (80.0)	108 (67.9)
	Not known		9 (5.8)	0 (0.0)	9 (5.7)
	Yes		41 (26.6)	1 (20.0)	42 (26.4)
	Missing		2	0	2
Horse handled by multiple persons	No		12 (7.7)	1 (25.0)	13 (8.2)
	Yes		143 (92.3)	3 (75.0)	146 (91.8)
	Missing		1	1	2
Other than riding/trotter/ breeding/working horse	No		141 (90.4)	5 (100.0)	146 (90.7)
	Yes		15 (9.6)	0 (0.0)	15 (9.3)
Riding horse	No		27 (17.3)	1 (20.0)	28 (17.4)
	Yes		129 (82.7)	4 (80.0)	133 (82.6)
Trotter	No		146 (93.6)	4 (80.0)	150 (93.2)
	Yes		10 (6.4)	1 (20.0)	11 (6.8)
Breeding horse	No		153 (98.1)	5 (100.0)	158 (98.1)
	Yes		3 (1.9)	0 (0.0)	3 (1.9)
Working horse	No		156 (100.0)	5 (100.0)	161 (100.0)
Known MRSA/ESBL carrier horse in close contact	No		120 (76.9)	4 (80.0)	124 (77.0)
	Not known		27 (17.3)	1 (20.0)	28 (17.4)
	Yes		9 (5.8)	0 (0.0)	9 (5.6)
Known MRSA/ESBL carrier person in close contact	No		141 (90.4)	4 (80.0)	145 (90.1)
	Not known		15 (9.6)	1 (20.0)	16 (9.9)
Has Cushing or IBD	No		144 (97.3)	4 (100.0)	148 (97.4)
	Yes		4 (2.7)	0 (0.0)	4 (2.6)
	Missing		8	1	9
Breed	Standardbred		15 (9.6)	1 (20.0)	16 (9.9)
	Other		24 (15.4)	2 (40.0)	26 (16.1)
	Pony		23 (14.7)	0 (0.0)	23 (14.3)
	Warmblood		69 (44.2)	2 (40.0)	71 (44.1)
	Finnhorse		25 (16.0)	0 (0.0)	25 (15.5)
Hospitalisation within three months	No		85 (54.5)	0 (0.0)	85 (52.8)
	Yes		71 (45.5)	5 (100.0)	76 (47.2)
Gender	Stallion		12 (7.7)	0 (0.0)	12 (7.5)
	Gelding		82 (52.6)	3 (60.0)	85 (52.8)
	Mare		62 (39.7)	2 (40.0)	64 (39.8)
Cat(s) on the home stable premises	No		63 (40.4)	1 (20.0)	64 (39.8)
	Yes		93 (59.6)	4 (80.0)	97 (60.2)
Dog(s) on the home stable premises	No		37 (23.7)	1 (20.0)	38 (23.6)
	Yes		119 (76.3)	4 (80.0)	123 (76.4)
Bird(s) on the home stable premises	No		138 (88.5)	5 (100.0)	143 (88.8)
	Yes		18 (11.5)	0 (0.0)	18 (11.2)
Other animals than cats/dogs/birds/cattle on the home stable premises	No		136 (87.2)	5 (100.0)	141 (87.6)
	Yes		20 (12.8)	0 (0.0)	20 (12.4)
Cattle on the home stable premises	No		154 (98.7)	5 (100.0)	159 (98.8)
	Yes		2 (1.3)	0 (0.0)	2 (1.2)
Type of home stable: riding school	No		134 (85.9)	4 (80.0)	138 (85.7)
	Yes		22 (14.1)	1 (20.0)	23 (14.3)
Type of home stable: private	No		30 (19.2)	1 (20.0)	31 (19.3)
	Yes		126 (80.8)	4 (80.0)	130 (80.7)
Attendance at an equine event within three months	No		118 (76.6)	4 (100.0)	122 (77.2)
	Not known		1 (0.6)	0 (0.0)	1 (0.6)
	Yes		35 (22.7)	0 (0.0)	35 (22.2)
	Missing		2	1	3
Urgency: outpatient	No		8 (5.1)	0 (0.0)	8 (5.0)
	Yes		148 (94.9)	5 (100.0)	153 (95.0)
Urgency: emergency service patient	No		148 (94.9)	5 (100.0)	153 (95.0)
	Yes		8 (5.1)	0 (0.0)	8 (5.0)
Muzzle contact possible at home stable	No		10 (6.4)	1 (20.0)	11 (6.8)
	Yes		146 (93.6)	4 (80.0)	150 (93.2)
Imported or visited abroad within three months	No		152 (97.4)	4 (80.0)	156 (96.9)
	Yes		4 (2.6)	1 (20.0)	5 (3.1)

Based on univariate analysis, antimicrobial treatment within three months (not known vs. no OR 48.61, 95% CI 2.84–833.76, *p* = 0.007; yes vs. no OR 8.46, 95% CI 1.19–60.36, *p* = 0.03), surgical procedure within three months (OR 10.48, 95% CI 1.89–58.27, *p* = 0.007), and imported or visited abroad within three months (OR 11.30, 95% CI 1.21–105.26, *p* = 0.03) were selected for multivariate analysis (selected variables in Table 4). None remained statistically significant (Table 4).

**Table 4:** Factors associated with harbouring ESBL-producing Enterobacterales on admission to the Equine Veterinary Teaching Hospital

Variables	ESBL-E cases (*n* = 5)		ESBL-E controls (*n* = 156)		Univariate logistic regression		Multivariate logistic regression	
**Continuous variable**	n	mean	n	mean	Unadjusted OR (95% CI)	Univariate *p*	Adjusted OR (95% CI)	Multivariate *p*
Age (years)	5	7.60	156	10.40	0.91 (0.77–1.08)	0.27		
Number of horses at home stable	5	25.20	153	19.41	1.02 (0.98–1.07)	0.34		
**Categorical variable**	n	%	n	%				
Antimicrobial treatment within three months						**0.02**		
Antimicrobial treatment within three months (not known vs. no)	1	20.00	2	1.33	48.62 (2.84–833.76)	**0.007**	21.72 (0.97–485.75)	0.052
Antimicrobial treatment within three months (yes vs. no)	3	60.00	33	21.20	8.46 (1.19–60.36)	**0.03**	4.14 (0.49–34.99)	0.192
Previously carrier of MRSA or ESBL						0.20		
Previously carrier of MRSA or ESBL (not known vs. no)	0	0.00	5	3.20	2.92 (0.11–79.79)	0.53		
Previously carrier of MRSA or ESBL (yes vs. no)	1	20.00	7	4.50	6.42 (0.80–51.62)	0.08		
Surgical procedure within three months (yes vs. no)	3	60.00	18	11.50	10.48 (1.89–58.27)	**0.007**	3.57 (0.51–24.98)	0.200
Horse handled by multiple persons (yes vs. no)	3	75.00	143	92.30	0.20 (0.03–1.59)	0.13		
Hospitalisation within three months (yes vs. no)	5	100.00	71	45.50	13.15 (0.70–246.17)	0.08		
Type of home stable: riding school (yes vs. no)	1	20.00	22	14.10	1.99 (0.29–13.78)	0.48		
Type of home stable: private (yes vs. no)	4	80.00	126	80.80	0.72 (0.11–4.91)	0.74		
Attendance at an equine event within three months (yes vs. no)	0	0.00	35	22.70	0.37 (0.02–7.34)	0.51		
Muzzle contact possible at home stable (yes vs. no)	4	80.00	146	93.60	0.22 (0.03–1.62)	0.14		
Imported or visited abroad within three months (yes vs. no)	1	20.00	4	2.60	11.30 (1.21–105.26)	**0.03**	7.54 (0.62–92.32)	0.114

Additional categorical variables in the risk factor analyses are presented in Table 5.

**Table 5:** Additional categorical variables for association with harbouring ESBL-producing Enterobacterales on admission to the Equine Veterinary Teaching Hospital

Variable	ESBL-E cases (*n* = 5)		ESBL-E controls (*n* = 156)		Univariate logistic regression	
	*n*	%	*n*	%	Unadjusted OR (95% CI)	Univariate *p*
Housed in other than individual stall/ open shed (yes vs. no)	0	0.00	7	4.50	1.81 (0.08–43.46)	0.71
Housed in open shed (yes vs. no)	0	0.00	22	14.10	0.54 (0.03–10.81)	0.69
Housed in individual stall (yes vs. no)	5	100.00	128	82.10	2.44 (0.13–47.67)	0.56
Regularly handled by a person working on a pig farm (not known vs. no)	0	0.00	2	1.30	5.47 (0.12–248.48)	0.38
Regularly handled by a person working on a pig farm (yes vs. no)	0	0.00	3	1.90	3.91 (0.12–132.92)	0.45
Regularly handled by a person working in healthcare (not known vs. no)	0	0.00	9	5.80	1.22 (0.05–28.35)	0.90
Regularly handled by a person working in healthcare (yes vs. no)	1	20.00	41	26.60	0.84 (0.13–5.62)	0.86
Other than riding/trotter/breeding horse (yes vs. no)	0	0.00	15	9.60	0.83 (0.04–17.20)	0.90
Riding horse (yes vs. no)	4	80.00	129	82.70	0.64 (0.09–4.35)	0.65
Trotter (yes vs. no)	1	20.00	10	6.40	4.65 (0.62–35.05)	0.14
Breeding horse (yes vs. no)	0	0.00	3	1.90	3.99 (0.12–135.55)	0.44
Known close contact with MRSA/ESBL carrier horse (not known vs. no)	1	20.00	27	17.30	1.46 (0.21–9.97)	0.70
Known close contact with MRSA/ESBL carrier horse (yes vs. no)	0	0.00	9	5.80	1.41 (0.06–32.66)	0.83
Known close contact with MRSA/ESBL carrier person (not known vs. no)	1	20.00	15	9.60	3.04 (0.43–21.77)	0.27
Has Cushing or IBD (yes vs. no)	0	0.00	4	2.70	3.57 (0.12–106.85)	0.46
Breed						0.63
Breed (trotting warmblood vs. riding warmblood)	1	20.00	15	9.60	2.69 (0.32–22.97)	0.37
Breed (other than trotting warmblood/pony/ Finnhorse vs. riding warmblood)	2	40.00	24	15.40	2.84 (0.45–17.80)	0.27
Breed (pony vs. riding warmblood)	0	0.00	23	14.70	0.59 (0.03–13.54)	0.74
Breed (Finnhorse vs. riding warmblood)	0	0.00	25	16.00	0.55 (0.02–12.40)	0.70
Gender						1.00
Gender (stallion vs. mare)	0	0.00	12	7.70	1.00 (0.04–24.68)	1.00
Gender (gelding vs. mare)	3	60.00	82	52.60	1.06 (0.20–5.62)	0.94
Cat(s) on the home stable premises (yes vs. no)	4	80.00	93	59.60	2.04 (0.31–13.47)	0.46
Dog(s) on the home stable premises (yes vs. no)	4	80.00	119	76.30	0.94 (0.14–6.32)	0.95
Bird(s) on the home stable premises (yes vs. no)	0	0.00	18	11.50	0.68 (0.03–13.81)	0.80
Animals other than cats/dogs/birds/cattle on the home stable premises (yes vs. no)	0	0.00	20	12.80	0.61 (0.03–12.15)	0.74
Cattle on the home stable premises (yes vs. no)	0	0.00	2	1.30	5.62 (0.12–255.06)	0.38
Urgency: outpatient (yes vs. no)	5	100.00	148	94.90	0.63 (0.03–14.60)	0.77
Urgency: emergency service patient (yes vs. no)	0	0.00	8	5.10	1.59 (0.07–36.82)	0.77

### Longitudinal excretion of ESBL-E

#### Description of the study population

Thirteen ESBL-E positive horses were enrolled in the longitudinal follow-up study, including the five from the prevalence study and eight horses from the discharge screening of ESBL-E that fulfilled the inclusion criteria.

Five horses completed the entire year of samplings, while eight horses were lost to follow-up (Additional file 4). Further specimens were not received from 2/13 horses (15%) after the initial ESBL-E positive specimen. None of the participating horses developed a clinical infection caused by ESBL-E during the sampling period.

#### Description of ESBL-E excretion

Most of the participating horses (8/13, 62%) were ESBL-E negative in all specimens obtained after the initial ESBL-E positive specimen (Additional file 4). One horse was persistent ESBL-E positive in two consecutive samplings (a period of four weeks). Two horses appeared ESBL-E positive again after having been ESBL-E negative in one or multiple specimens (minimum and maximum time interval testing ESBL-E negative between ESBL-E positive specimens 11 weeks and 21 weeks, respectively).

The ESBL-E isolates of the horses recruited in the study and the isolates that were obtained after the initial specimen are presented in Table 1. Horse no. 2 shed phenotypically identical *K. pneumoniae* isolate on week 4 as initially (Table 1, Additional file 4). Horse no. 7 was ESBL-E negative on week 9, but again ESBL-E positive on week 11 (after hospital visit and antibiotic treatment) shedding a phenotypically different *E. cloacae* isolate than on week 0. Horse no. 10 appeared ESBL-E negative in four consecutive specimens after week 0, but on week 21 shed *E. coli* (initially *E. cloacae*). The *E. coli* isolate in the subsequent specimen on week 26 was phenotypically identical.

## Discussion

This is the first study conducted on ESBL-E prevalence in horses in Finland. The low prevalence of ESBL-E (3%; 95% CI 1–7%) in horses admitted to an equine hospital is consistent with our hypothesis, as we expected the result to reflect the prevalence in other species in Finland. None of the tested variables were statistically significant for association with ESBL-E colonisation in horses on admission to an equine hospital. The ESBL-E species observed in our study reflected previous research findings on horses. There was good concordance between antimicrobial resistance phenotype and genotype. We present here a novel sequence type of *K. pneumoniae* (ST6179), which was likely causative of clonal spread at an equine teaching hospital based on epidemiological and molecular data. In horses enrolled in the one-year follow-up study, ESBL-E were unlikely to be isolated after the initial ESBL-E positive specimen.

As the low prevalence was expected, we decided to use pre-culture enrichment to increase the sensitivity of detecting ESBL-E in the specimens and we also wanted to investigate any ESBL-E isolates appearing in horses in Finland regardless of the magnitude of shedding. In other countries, the prevalence has indeed been higher. In Germany, the colonisation rate on admission to an equine hospital was 11% (no enrichment used); the rate was 20% in Israel (enrichment used) [1, 2]. To date, prevalence data from the Nordic countries are lacking. We suspect that the low prevalence in our study might be partly due to the prudent use of antimicrobials in animals in Finland [22] and partly because Finland is in Northern Europe, with lower intercountry mobility.

The sample population in our prevalence and risk factor study consisted mainly of outpatients living in the greater metropolitan area of Helsinki. Thus, the prevalence cannot be readily generalised to the equine population of Finland, especially since not all the enrolled horses were healthy. However, the study population is representative of the patient material of the EVTH. Since most of the equine population is concentrated in Southern Finland and there is likely more frequent movement between premises, the prevalence of ESBL-E might be even lower in horses from other parts of Finland.

The *K. pneumoniae* isolates harboured resistance genes for multiple classes of antimicrobials and were co-resistant to fluoroquinolones, sulfamethoxazole-trimethoprim, tetracyclines, and gentamicin. The most common ESBL gene families in horses are CTX-M and SHV, and particularly the genes *bla*_CTX−M−1_, *bla*_CTX−M−15_, and *bla*_SHV−12_ [2, 4, 9, 17, 23–26]. This is consistent with the findings of our study, as the *K. pneumoniae* isolates harboured *bla*_CTX−M−15_ and two of the *E. coli* isolates had the *bla*_CTX−M−1_ gene. The broad-spectrum β-lactamase encoding gene *bla*_TEM−206_ has been reported among *K. pneumoniae* isolates in urban riverine environment in India [27] and in a neonatal intensive care unit in Italy [28]. Our study is the first to report this gene in *K. pneumoniae* in animals. The gene has been detected in *E. coli* in livestock, specifically in pig farms, cattle, and chickens [29–31]. It appears that the gene can adapt to genomes of different bacterial species in various hosts and spread effectively in the animal community and the environment.

Two of the *K. pneumoniae* isolates were indistinguishable by core-genome multi-locus sequence typing (cgMLST), and the third isolate was also clonally related as it differed only by three alleles (out of 2358). These findings and the recent concurrent hospitalisation indicate clonal spread of ESBL-E and nosocomial transmission at the EVTH. One case report on suspected nosocomial infections caused by ESBL-E in a German equine hospital has been published [32]. In Israel, it was observed that ESBL-producing *Salmonella enterica* spread clonally between seven horses at a veterinary equine teaching hospital [33]. These discoveries underline the importance of the equine hospital setting as a potential risk environment for spread of ESBL-E. Horse faeces likely contaminate the environment, and selection pressure is often increased due to antimicrobial treatments. Furthermore, hospitalised horses are usually more susceptible to infections, and multiple persons are treating patient horses simultaneously. It is alarming that ESBL-E strains with extensive drug resistance characteristics are circulating in the equine population, as few antimicrobial substances are suitable and available for use in horses.

We also detected the presence of type 3 fimbrial gene cluster (*mrk*) in the *K. pneumoniae* isolates, and these fimbriae may promote colonisation and attachment to host cells in a horse and biofilm formation [34, 35]. For example, this gene cluster has been detected in a clinical wound infection isolate of *K. pneumoniae* ST1228 from a horse in Austria [4], which suggests that the isolates in our study may cause infections in horses even though they were discovered from rectal screening specimens. This finding is worrisome, especially considering that nosocomial spread of these multi-resistant bacteria was suspected.

The discovered ST1679 clone belongs to the same clonal group CG307 as a commonly recognised high-risk clone *K. pneumoniae* ST307 [4, 36, 37]. A CTX-M-15-associated strain of ST307 caused an outbreak involving clinical manifestations at the EVTH in 2014 [3]. As ST1679 only differs from ST307 by one allele in MLST, they are genetically very similar, which leads us to the question if there has been an ongoing evolution of ESBL-producing *K. pneumoniae* at the EVTH throughout the years. The outbreak clone ST307 expressed an identical phenotypical resistance pattern as the ST1679 clone in the present study. However, detailed molecular comparison is lacking as the ST307 clone at the EVTH has not been subjected to whole-genome sequencing. To gain knowledge on plasmid evolution and its possible connection to equine patients, further genomic investigations in the subject, specifically plasmid analyses, are warranted.

In the risk factor study, none of the tested variables were statistically associated with ESBL-E colonisation on admission, which is likely be at least partly due to the low prevalence compared to the sample size. Furthermore, the study was conducted during the COVID-19 pandemic and during winter. In Finland there are fewer equine events organised during the winter due to lack of indoor facilities. Had the study been conducted in summer, an increase in the number of equine contacts could potentially have affected the results.

However, antimicrobial treatment, surgical operation, or visiting abroad (or import) within three months were statistically significant in univariate analysis. This provides some indication to their relevance as factors to consider when establishing a risk class for a patient admitted to an equine hospital. Antimicrobial treatment is associated with multidrug-resistant *E. coli* or ESBL-E colonisation in previous studies [1, 38]. Horses undergoing surgical treatment commonly also receive antimicrobials, which leads to an association between these factors and thus not ending up as independent risk factors in our study. Nevertheless, we suggest that the aforementioned factors should be considered when estimating the risk of ESBL-E colonisation of an admitted equine patient. It is likely that some of the variables could have become statistically significant with increased power. It should be noted that the CIs of the ORs were broad due to the rarity of ESBL-E positive horses.

Even though the longitudinal study of individual horses colonised with ESBL-E was descriptive in nature, the results of our study indicate that it is unlikely that ESBL-E isolates persist in horses for a long time, and the exposure risk in the equine community is estimated to be minimal. Most of the horses remained ESBL-E negative after the initial specimen. However, it is noteworthy that the excretion of ESBL-E in dogs is highly dynamic, which is a complicating finding from epidemiological and infection control perspectives, as it makes an animal’s ESBL-E status less predictable [19]. A study conducted in the UK showed that the odds of resistance in *E. coli* diminish approximately two weeks after antimicrobial treatment in non-hospitalised horses [18]. Another UK study indicated that being stabled in the same yard as a horse that has recently been hospitalised increased the risk of being colonised with ESBL *E. coli* [38]. It may be worthwhile to consider if ESBL-E positive horses should be managed separately for some weeks at home stables. However, more investigations on the dynamics of ESBL-E over time in healthy horses are needed, as healthy horses at home stable are not subject to the same infection pressure as patients in equine hospitals.

Many of the horses in the longitudinal study visited an equine clinic or hospital several times during the sampling period. Horse number 10 was initially colonised with ESBL *E. cloacae* (P-2771) and tested ESBL-E negative for several months but tested ESBL-E positive again in week 21. However, the bacterial species was different, this time being ESBL *E. coli* (HE-14). The horse visited an equine hospital between samplings, which raises the possibility that the horse was recolonised. This is the most likely explanation, as the strain did not appear in the previous enriched specimens and the horse did not receive antimicrobials before the new ESBL-E positive specimen, which could have created selection pressure. However, the recolonisation could mask the shedding of the initial ESBL-E strain (P-2771) and thus complicate the interpretation of the study.

There were some limitations in the execution of the longitudinal study. The sample population was particularly small due to limited resources and many horses were lost to follow-up. Thus, no statistical analyses were performed. Recall bias should also be considered, as we interviewed the owners on the antimicrobial treatments and hospital visits only after the study was finished. To achieve accurate records, we could have conducted a phone interview after each sampling or created a form and ask the owner to send to the laboratory with the specimen.

Knowledge on the local prevalence and risk factors for ESBL-E carriage in horses is crucial to establish targeted infection control programs in equine settings. As ESBL-E can spread effectively in the equine community, we encourage equine veterinarians and scientists to collect more epidemiological and molecular data and to conduct further investigations on the dynamics of ESBL-E in equine hospitals and clinics utilising whole-genome sequencing. Rapid sequencing could aid in identifying the source of nosocomial spread or the magnitude of the spread of certain strains in outbreaks, as well as in monitoring persistence of (resistant) pathogens. A deeper understanding on the spread of ESBL genes and bacteria will allow for sufficient infection control measures and improved short- and long-term surveillance to reduce the risk of nosocomial transmission. Routine sequencing of pathogens is still not standard practice in most equine practices but could be implemented in active and passive surveillance strategies along with phenotypic susceptibility analysis. Sharing the best practices of designing and implementing infection control programs at equine clinics or hospitals could benefit the broader equine medicine community and finally the equine patients at clinics and hospitals. Information from the last three months on antimicrobial treatments, surgical treatments, and international travel are suggested to be recorded for patients entering equine hospitals to evaluate the need for preventative actions.

## Conclusion

The prevalence of ESBL-E in horses visiting a veterinary teaching hospital in Finland was low, which suggests a low national prevalence among horses in Finland. While no statistically significant risk factors were identified, previous antimicrobial treatment was implicated as a possible risk factor. The situation is thus overall favourable, but equine practitioners should adhere to antimicrobial stewardship guidelines to maintain or improve the situation. As nosocomial transmission of ESBL-E was suspected in three horses, the importance of infection prevention and control at equine hospitals and clinics should be emphasized. Horses seem to carry ESBL-producing bacteria only for a short time, but hospital visits and antimicrobial treatment may prolong carriage or expose the horse to other ESBL-producing bacteria.

## Methods

### Study design, study population and sample size

#### Prevalence and risk factor study

This was a cross-sectional prevalence study on equine patients entering the EVTH between October 2020 and April 2021 that were sampled for asymptomatic ESBL-E carriage. The EVTH is the only equine teaching hospital in Finland and receives slightly under 3000 patients per year. The hospital treats both primary (from the metropolitan area) and referral equine patients (nationwide).

Additionally, after obtaining research consent, the person accompanying the horse was presented with a horse-focused questionnaire based on previous literature on possible risk factors for ESBL-E colonisation (Additional file 3); putative risk factors for the analysis included the signalment of the horse, factors concerning the hospital visit, characteristics of the home stable and the horse’s close contacts, visits outside the home stable, as well as recent procedures and medications. The questionnaire was tested prior to the start of the study by horse-owners employed by the EVTH to ensure that the questions and concepts were understood as meant and that all necessary response alternatives were covered. As the test was successful, the original questionnaire was used without modifications. The person accompanying the equine patient was interviewed by KE, EAK, or a trained licentiate thesis student. If there was no person present with the patient, the owner was contacted by phone to inquire for research consent and to present the questionnaire. Study participation was voluntary.

The study population consisted of a convenience sample of equine patients entering the EVTH. Both outpatients and emergency service patients of all ages, breeds, and sexes were included. Horses entering the hospital during off-hours (outside Monday to Friday 8–16, or on national holidays), those that were not given research consent, or both, were excluded from the study.

The sample size was estimated with EpiTools (https://epitools.ausvet.com.au/oneproportion). In 2018, there were approximately 74 400 horses in Finland [39]. With a prevalence estimate of 5% and desired precision of 3%, a sample size of 200 horses was calculated.

#### Longitudinal study

A prospective observational study with a study period of one year was designed based on literature on ESBL-E shedding in animals and available personnel resources [17–20]. The ESBL-E positive horses of the prevalence study were enrolled in the longitudinal study. As an epidemiological investigation was initiated at the EVTH due to phenotypically similar findings in the prevalence study, patient screening at discharge was launched to reveal possible ESBL-E positive hospitalised horses. To increase the number of participating horses in the longitudinal study, a separate convenience sample of horses that were ESBL-E positive at the discharge screening were included, in addition to the horses from the prevalence study. The inclusion criteria were (1) rectal colonisation of the horse with ESBL-E, (2) consent for participating in the study from the owner, and (3) absence of active clinical infection caused by any ESBL-E at the time of enrolment.

The sampling scheme started from the sampling date of the initial ESBL-E positive specimen (i.e., the starting date was unique to all participating horses). For the first six months, specimens were asked to be obtained every month, resulting in six specimens. After the first six months, two additional specimens were to be obtained three months apart (i.e., nine months and twelve months after the initial sampling date).

A phone interview with the owner was conducted once the study period was finished to record possible antimicrobial treatments and hospital visits of the horse during the study period.

#### Ethical review

The study was approved by the Viikki Campus Research Ethics Committee of the University of Helsinki (statement no. 10/2020). The guidelines of the Finnish Advisory Board on Research Integrity for good scientific practice were followed in the study design [40].

#### Collection of ESBL-E specimens

Bacteriological specimens were obtained from the rectum of the horse using a sterile cotton swab (M40 Transystem™ with Amies gel, Copan Italia S.p.a., Italy). Persons obtaining the specimens were trained by KE or EAK and given written instructions.

For the prevalence study, the sampling was performed on admission without delay and before any procedures.

For the longitudinal study, the owners of the ESBL-E positive horses in the one-year follow-up study were trained for rectal swab sampling and additionally received written instructions. A sampling kit of cotton swabs and material for shipping the specimens to the Clinical Microbiology Laboratory (CML) of the Veterinary Faculty of the University of Helsinki was either handed over or sent to the owner. The shipping was instructed to be performed without delay for the specimen to arrive at the laboratory within 48 h from the sampling. Although the owners were reminded of obtaining the follow-up specimens, the acquisition of them was dependent on the owner’s willingness to continue in the study.

### Microbiological methods

#### Bacterial culturing

Bacterial specimens were cultured at the CML. The specimen was first placed in 3 ml of buffered peptone water (Oxoid Ltd., UK), enriched at 36 ± 2 °C overnight, and then plated onto ESBL selective growth medium (Brilliance™ ESBL Agar, Oxoid Deutschland GmbH, Germany). The agar plates were incubated at 36 ± 2 °C and interpreted at 24 and 48 h.

#### Species identification and susceptibility testing

Suspected ESBL-E colonies were identified using matrix-assisted laser desorption/ionisation time-of-flight (MALDI-TOF) mass spectrometry (Bruker MALDI Biotyper Microflex LT, Bruker Daltonik GmbH, Germany). A score value ≥ 2.0 was considered as highly confident identification. Confirmed ESBL-E species were subcultured onto tryptone soya agar with sheep blood (Oxoid Deutschland GmbH, Germany), grown at 36 ± 2 °C for 24 h.

The isolates were tested for antimicrobial susceptibility on Mueller-Hinton agar (Oxoid Ltd., UK). The standard laboratory disc panel for gram-negative species consisted of ampicillin (10 µg), amoxicillin/clavulanic acid (30 µg), cefpodoxime (10 µg), meropenem (10 µg), enrofloxacin (5 µg), sulfamethoxazole-trimethoprim (25 µg), amikacin (30 µg), gentamicin (10 µg), chloramphenicol (30 µg), tetracycline (20 µg), and doxycycline (30 µg) (Oxoid Ltd., UK). Susceptibility for colistin was investigated using Colistin ETEST^®^ (bioMérieux SA, France). The CLSI disc diffusion guidelines were followed (tetracycline [41], colistin [42], the rest of the panel [43, 44]). In addition, phenotypic identification of ESBL production was performed using double-disc diffusion test (including cefotaxime 30 µg, cefotaxime/clavulanic acid 30/10 µg, ceftazidime 30 µg, and ceftazidime/clavulanic acid 30/10 µg) and MASTDISCS^®^ Combi (Mast Group Ltd., UK). *E. coli* NCTC 13351 and *E. coli* ATCC 25922 were used as ESBL-positive and ESBL-negative quality control strains, respectively, and for the quality control of the standard disc diffusion panel. The ESBL-E isolates were frozen in skim milk at −80 °C until further investigation.

#### DNA extraction and whole-genome sequencing

The DNA of the ESBL-E isolates in the prevalence study was extracted and purified using DNeasy^®^ Blood & Tissue Kit (QIAGEN GmbH, Germany). The pre-treatment was performed according to the manufacturer’s instructions for gram-negative bacteria. The remainder of the preparation protocol was performed according to Purification of Total DNA from Animal Tissues (SpinColumn Protocol) in the user handbook. The extraction was automated using a QIAcube^®^ nucleic acid extraction unit (QIAGEN GmbH, Germany). DNA quantity was measured with an Invitrogen Qubit™ 4 fluorometer (Life Technologies Holdings Pte Ltd., Singapore) and quality with a NanoDrop™ spectrophotometer (NanoDrop Technologies, USA; for HE-6, HE-8, HE-15) and DeNovix DS-11+ (DeNovix Inc., USA; for HE-3, HE-4, HE-5). DNA was eluted in Buffer AE (QIAGEN GmbH, Germany).

Whole-genome sequencing was performed by Novogene (UK) Company Limited. The NEBNext^®^ Ultra™ DNA Library Prep Kit (New England BioLabs, USA) was used for library preparation. Sequencing was performed using Illumina NovaSeq 6000 platform (Illumina, Inc., USA) to generate paired-end 150 bp reads.

The raw reads of *E. coli* were deposited into the European Nucleotide Archive (ENA). Accession numbers of the isolates are provided in Table 1 (project accession number PRJEB71437).

#### Bioinformatics

Raw reads were processed in Ridom SeqSphere+ (software version 7.7.5, Ridom GmbH, Germany) with default parameters, including data quality assessment with FastQC (version 0.11.7) [45], trimming of adapters with Trimmomatic (version 0.36) [46], and de-novo assembly with SKESA (version 2.3.0) [47]. Statistics of the assembly phase are presented in Additional file 5.

Bioinformatic analyses were performed on the assembled sequences using web-based tools (Center for Genomic Epidemiology (CGE), DTU, Denmark) and in Ridom SeqSphere + with default settings. Antimicrobial resistance genes were identified with NCBI AMRFinderPlus (version 3.2.3) [48] and in ResFinder (version 4.4.1) [49, 50], plasmid replicons in PlasmidFinder (version 2.1) [51], and virulence genes in VirulenceFinder (for *E. coli*, version 2.0.3) [52, 53] and with VFDB (for *K. pneumoniae*, version 2020-Feb-28, http://www.mgc.ac.cn/VFs/). Serotyping of *E. coli* was run on SeroTypeFinder by CGE (version 2.0.1) [54] and phylogrouping of *E. coli* was performed on the web-based ClermonTyping tool [55].

Multi-locus sequence typing (MLST) was performed in MLST (version 2.0) by CGE [56]. The sequences of *K. pneumoniae* were submitted to the Institut Pasteur MLST database (https://bigsdb.pasteur.fr/klebsiella) for assignment of novel sequence types (accession numbers provided in Table 1).

For cgMLST producing a minimum spanning tree in Ridom SeqSphere+, the *K. pneumoniae* genomes were mapped using the ST307 reference strain NR5632 (GenBank accession no. CP025143) in *K. pneumoniae* sensu lato cgMLST (version 1.0). The cutoff value for relatedness in *K. pneumoniae* was set to ≤ 10 alleles (57,58).

#### Epidemiological data

Epidemiological (patient) data were obtained from the patient information system of the EVTH (Provet Net, Nordhealth Finland Oy, Finland) and combined with laboratory data of the CML within the same information system.

#### Statistical analyses

The 95% confidence interval (CI) for the prevalence estimate was calculated using an EpiTools calculator (https://epitools.ausvet.com.au/ciproportion) with Wilson score method due to low prevalence estimate.

The questionnaire data were pre-processed in Microsoft Excel by KE and EAK and analysed by 4Pharma Oy (Finland). Statistical analyses were performed using SAS System for Windows (version 9.4, SAS Institute Inc., Cary, NC, USA). A control was defined with the outcome “ESBL-E negative” and a case with “ESBL-E positive” in the screening specimen.

Each risk factor for ESBL-E colonisation was first analysed individually using Firth’s logistic regression. This method was chosen due to extremely rare ESBL-E cases to minimise the analytical bias caused by rare events. Following the univariate analyses, a multivariate model was constructed.

The multivariate model was constructed in a stepwise manner, including the risk factors in the selection process that had p-values (Wald) < 0.05 in univariate modelling. The variable selection was performed using a traditional implementation of stepwise selection. The Significance Level for Entry (SLE) was set to 0.15 and the Significance Level to Stay (SLS) was set to 0.20. If any effect at any step in the model was not significant at the SLE, then the least significant of the effects was removed from the model, and the algorithm proceeded to the next step. After necessary deletions, another effect, whose addition yielded the most significant F value, was added to the model, and the algorithm proceeded to the next step. The stepwise process ended when none of the effects outside the model had an F statistic significant at the SLE and every effect in the model was significant at the SLS. The stepwise selection was performed using the GLMSELECT procedure in SAS.

Multivariate analysis was performed using Firth’s logistic regression. Odds ratios (OR) with 95% CIs were calculated.

## Electronic supplementary material

Below is the link to the electronic supplementary material.

**Additional file 1**. Antimicrobial resistance genes of the extended-spectrum β-lactamase -producing Enterobacterales (ESBL-E) isolates in the prevalence study

**Additional file 2**. Virulence genes of the extended-spectrum β-lactamase -producing Enterobacterales (ESBL-E) isolates in the prevalence study

**Additional file 3**. Questionnaire (translated from Finnish to English) for determining risk factors for extended-spectrum β-lactamase -producing Enterobacterales (ESBL-E) carriage in admitted horses at the Equine Veterinary Teaching Hospital

**Additional file 4**. Excretion of extended-spectrum β-lactamase -producing Enterobacterales (ESBL-E) in horses recruited in a one-year (52 weeks) follow-up study

**Additional file 5**. Statistics of the de-novo assembly of the extended-spectrum β-lactamase -producing Enterobacterales (ESBL-E) isolates in the prevalence study

## Data Availability

The raw reads generated during the current study are available in the European Nucleotide Archive (ENA) at EMBL-EBI under study accession number PRJEB71437 (https://www.ebi.ac.uk/ena/browser/view/PRJEB71437) and in the Institut Pasteur MLST database (https://bigsdb.pasteur.fr/klebsiella). The isolate accession numbers are provided in Table 1.

## Declarations

### Prior publication

Parts of the preliminary results of the study (prevalence of ESBL-E on admission, whole-genome sequencing of *K. pneumoniae*) have been presented at the poster sessions of the 13th International Meeting on Microbial Epidemiological Markers (IMMEM XIII), Bath, United Kingdom [59] and Finnish Veterinary Congress 2022, Helsinki, Finland [60] and published in a non-peer-reviewed licentiate thesis project (in Finnish) related to veterinary studies (prevalence of ESBL-E on admission, questionnaire) [61].

## Abbreviations

cgMLST: Core-genome multi-locus sequence typing
ESBL: Extended spectrum β-lactamase
ESBL-E: Extended spectrum β-lactamase -producing Enterobacterales
MLST: Multi-locus sequence typing
MRSA: Methicillin resistant Staphylococcus aureus
