# Supplementary material for: Prevalence, risk factors, and characterisation of extended-spectrum β-lactamase -producing Enterobacterales (ESBL-E) in horses entering an equine hospital and description of longitudinal excretion
Source: BMC Vet Res. 2024 Sep 13;20:412. doi: 10.1186/s12917-024-04260-z (PMC11396584; doi:10.1186/s12917-024-04260-z)
Supplement: Supplementary file 1 — Additional file 1. Antimicrobial resistance genes of the extended-spectrum β-lactamase -producing Enterobacterales (ESBL-E) isolates in the prevalence study [file 12917_2024_4260_MOESM1_ESM.docx]

**Additional file 1.** Antimicrobial resistance genes of the extended-spectrum β-lactamase-producing Enterobacterales (ESBL-E) isolates in the prevalence study.

Blue cells indicate presence of gene and gray cells indicate absence of gene.

^1^ResFinder (CGE); ^2^NCBI AMRFinder.

|  |  | *Escherichia coli* | | | *Klebsiella pneumoniae* | | |
| --- | --- | --- | --- | --- | --- | --- | --- |
|  |  | HE-3 | HE-4 | HE-5 | HE-6 | HE-8 | HE-15 |
| β-lactam (*bla*) | EC^2^ |  |  |  |  |  |  |
|  | CTX-M-1^1,2^ |  |  |  |  |  |  |
|  | CTX-M-14^1,2^ |  |  |  |  |  |  |
|  | CTX-M-15^1,2^ |  |  |  |  |  |  |
|  | TEM-1B^1,2^ |  |  |  |  |  |  |
|  | TEM-206^1,2^ |  |  |  |  |  |  |
|  | SHV-28^1,2^ |  |  |  |  |  |  |
|  | SHV-106^1^ |  |  |  |  |  |  |
|  | OXA-1^1,2^ |  |  |  |  |  |  |
|  | OXA-10^1,2^ |  |  |  |  |  |  |
| Folate pathway antagonist Quinolone Amphenicol | OqxA^1,2^ |  |  |  |  |  |  |
|  | OqxB^1^ |  |  |  |  |  |  |
|  | OqxB19^2^ |  |  |  |  |  |  |
|  | dfrA14^1,2^ |  |  |  |  |  |  |
|  | dfrA17^1,2^ |  |  |  |  |  |  |
|  | sul1^1,2^ |  |  |  |  |  |  |
|  | sul2^1,2^ |  |  |  |  |  |  |
|  | aac(6')-Ib-cr^1^ |  |  |  |  |  |  |
|  | aac(6')-Ib-cr5^2^ |  |  |  |  |  |  |
|  | qnrB1^1,2^ |  |  |  |  |  |  |
|  | qnrB19^2^ |  |  |  |  |  |  |
|  | cmlA1^1^ |  |  |  |  |  |  |
|  | cmlA5^2^ |  |  |  |  |  |  |
|  | catA1^1,2^ |  |  |  |  |  |  |
|  | catB3^1^ |  |  |  |  |  |  |
| Aminoglycoside | aadA1^1,2^ |  |  |  |  |  |  |
|  | aadA5^1,2^ |  |  |  |  |  |  |
|  | aac(3)-IIa^1,2^ |  |  |  |  |  |  |
|  | aac(3)-IId^1,2^ |  |  |  |  |  |  |
|  | aph(3'')-Ib^1,2^ |  |  |  |  |  |  |
|  | aph(6)-Id^1,2^ |  |  |  |  |  |  |
| Tetracycline | tet(A)^1,2^ |  |  |  |  |  |  |
|  | tet(B)^1,2^ |  |  |  |  |  |  |
| Macrolide | mph(A)^1,2^ |  |  |  |  |  |  |
| Fosfomycin | fosA^2^ |  |  |  |  |  |  |
|  | fosA6^1^ |  |  |  |  |  |  |
| Rifamycin | ARR-2^1,2^ |  |  |  |  |  |  |
