# Supplementary material for: Prevalence, risk factors, and characterisation of extended-spectrum β-lactamase -producing Enterobacterales (ESBL-E) in horses entering an equine hospital and description of longitudinal excretion
Source: BMC Vet Res. 2024 Sep 13;20:412. doi: 10.1186/s12917-024-04260-z (PMC11396584; doi:10.1186/s12917-024-04260-z)
Supplement: Supplementary file 2 — Additional file 2. Virulence genes of the extended-spectrum β-lactamase -producing Enterobacterales (ESBL-E) isolates in the prevalence study [file 12917_2024_4260_MOESM2_ESM.docx]

**Additional file 2.** Virulence genes of the extended-spectrum β-lactamase-producing Enterobacterales (ESBL-E) isolates in the prevalence study.

Blue cells indicate the presence of a gene, and gray cells indicate the absence of a gene. Only genes detected in at least one isolate are shown. Genes marked with an asterisk were detected by VirulenceFinder (CGE) in addition to VFDB (in Ridom SeqSphere+).

| Virulence gene | *Escherichia coli* | | | *Klebsiella pneumoniae* | | |
| --- | --- | --- | --- | --- | --- | --- |
|  | HE-3 | HE-4 | HE-5 | HE-6 | HE-8 | HE-15 |
| acrA |  |  |  |  |  |  |
| acrB |  |  |  |  |  |  |
| air* |  |  |  |  |  |  |
| aslA |  |  |  |  |  |  |
| chuA |  |  |  |  |  |  |
| chuS |  |  |  |  |  |  |
| chuT |  |  |  |  |  |  |
| chuU |  |  |  |  |  |  |
| chuV |  |  |  |  |  |  |
| chuW |  |  |  |  |  |  |
| chuX |  |  |  |  |  |  |
| chuY |  |  |  |  |  |  |
| clpV tssH |  |  |  |  |  |  |
| cpsACP |  |  |  |  |  |  |
| csgA* |  |  |  |  |  |  |
| dotU tssL |  |  |  |  |  |  |
| eilA* |  |  |  |  |  |  |
| entA |  |  |  |  |  |  |
| entB |  |  |  |  |  |  |
| entC |  |  |  |  |  |  |
| entD |  |  |  |  |  |  |
| entE |  |  |  |  |  |  |
| entF |  |  |  |  |  |  |
| entS |  |  |  |  |  |  |
| espL1 |  |  |  |  |  |  |
| espL4 |  |  |  |  |  |  |
| espX1 |  |  |  |  |  |  |
| espX2 |  |  |  |  |  |  |
| espX4 |  |  |  |  |  |  |
| espX5 |  |  |  |  |  |  |
| espY2 |  |  |  |  |  |  |
| espY4 |  |  |  |  |  |  |
| fdeC* |  |  |  |  |  |  |
| fepA |  |  |  |  |  |  |
| fepB |  |  |  |  |  |  |
| fepC |  |  |  |  |  |  |
| fepD |  |  |  |  |  |  |
| fepG |  |  |  |  |  |  |
| fes |  |  |  |  |  |  |
| fimA |  |  |  |  |  |  |
| fimB |  |  |  |  |  |  |
| fimC |  |  |  |  |  |  |
| fimD |  |  |  |  |  |  |
| fimE |  |  |  |  |  |  |
| fimF |  |  |  |  |  |  |
| fimG |  |  |  |  |  |  |
| fimH |  |  |  |  |  |  |
| fimI |  |  |  |  |  |  |
| fimK |  |  |  |  |  |  |
| galF |  |  |  |  |  |  |
| glf |  |  |  |  |  |  |
| gnd |  |  |  |  |  |  |
| hcp tssD |  |  |  |  |  |  |
| hlyE* |  |  |  |  |  |  |
| icmF tssM |  |  |  |  |  |  |
| impA tssA |  |  |  |  |  |  |
| iroE |  |  |  |  |  |  |
| iss* |  |  |  |  |  |  |
| kfoC |  |  |  |  |  |  |
| lpfA* |  |  |  |  |  |  |
| mrkA |  |  |  |  |  |  |
| mrkB |  |  |  |  |  |  |
| mrkC |  |  |  |  |  |  |
| mrkD |  |  |  |  |  |  |
| mrkF |  |  |  |  |  |  |
| mrkH |  |  |  |  |  |  |
| mrkI |  |  |  |  |  |  |
| mrkJ |  |  |  |  |  |  |
| neuC |  |  |  |  |  |  |
| nlpI* |  |  |  |  |  |  |
| ompA |  |  |  |  |  |  |
| rcsA |  |  |  |  |  |  |
| rcsB |  |  |  |  |  |  |
| sciN tssJ |  |  |  |  |  |  |
| terC* |  |  |  |  |  |  |
| tli1 |  |  |  |  |  |  |
| tssF |  |  |  |  |  |  |
| tssG |  |  |  |  |  |  |
| ugd |  |  |  |  |  |  |
| vasE tssK |  |  |  |  |  |  |
| vgrG tssI |  |  |  |  |  |  |
| wbbM |  |  |  |  |  |  |
| wbbN |  |  |  |  |  |  |
| wbbO |  |  |  |  |  |  |
| wzi |  |  |  |  |  |  |
| wzm |  |  |  |  |  |  |
| wzt |  |  |  |  |  |  |
| ybdA |  |  |  |  |  |  |
| yehA* |  |  |  |  |  |  |
| yehB* |  |  |  |  |  |  |
| yehC* |  |  |  |  |  |  |
| yehD* |  |  |  |  |  |  |
