# Supplementary material for: Prevalence, risk factors, and characterisation of extended-spectrum β-lactamase -producing Enterobacterales (ESBL-E) in horses entering an equine hospital and description of longitudinal excretion
Source: BMC Vet Res. 2024 Sep 13;20:412. doi: 10.1186/s12917-024-04260-z (PMC11396584; doi:10.1186/s12917-024-04260-z)
Supplement: Supplementary file 3 — Additional file 3. Questionnaire (translated from Finnish to English) for determining risk factors for extended-spectrum β-lactamase -producing Enterobacterales (ESBL-E) carriage in admitted horses at the Equine Veterinary Teaching Hospital [file 12917_2024_4260_MOESM3_ESM.docx]

**Additional file 3.** Questionnaire (translated from Finnish to English) for determining risk factors for extended-spectrum β-lactamase-producing Enterobacterales (ESBL-E) carriage in admitted horses at the Equine Veterinary Teaching Hospital.

**Research Project - Antibiotic-resistant Bacteria in Horses**

The aim of the research project is to investigate the prevalence of antibiotic-resistant bacteria in horses in Finland and the transmission of these bacteria between horses in stable conditions. The study aims to identify risk factors for becoming a carrier and the duration of carriage. The research will be conducted at the Department of Equine and Small Animal Medicine of the University of Helsinki's Faculty of Veterinary Medicine.

The occurrence of antibiotic-resistant bacteria in both humans and animals has increased in recent years. Many of these bacteria are zoonotic, meaning they can transfer between animals and humans. Common examples include methicillin-resistant *Staphylococcus aureus* (MRSA) and extended-spectrum beta-lactamase-producing (ESBL) enteric bacteria. These bacteria are often resistant to multiple antibiotic groups, complicating the treatment of infections in both humans and animals.

MRSA and ESBL-producing bacteria can cause outbreaks in healthcare settings for both humans and animals worldwide. While the spread of these bacteria among animals and from animals to humans is well-documented in hospital environments, there is limited research on how these bacteria transfer among horses in home settings and the prevalence of such bacteria in horses living in Finland. Additionally, information on the duration of carriage is scarce. Our research project aims to address these questions.

We request mucosal swabs and rectal samples from your horse for this study. The sampling process does not cause pain or discomfort for the horse and is done upon the horse's arrival at the hospital. Participation in the study is possible for patients coming to the hospital during office hours.

If your horse is identified as a carrier of MRSA or ESBL in the study, we invite you to participate in a 1-year follow-up study. This sub-study aims to determine how long the horse carries the bacteria. Samples will be taken at the home stable monthly for the first 6 months and later every 3 months for the remaining 6 months. Duration of carriage has not been extensively studied in horses, making this information valuable.

If you wish to receive information about your horse's MRSA or ESBL carriage status after the study, please provide your email address on the form.

The information provided on the form will be treated confidentially. You can withdraw from the study at any time by contacting the responsible investigator, and any collected data will be destroyed.

Research Data Privacy Statement (*link provided*)

Responsible investigator (*contact information*)

**Consent to Donate Horse Samples for Research**

□ I consent to the use of my horse's information and samples for research purposes.

**Contact information**

Name: _____________________________

Email (Note: Complete this section if you want information about your horse's research results after the study.): _____________________________

Phone number: _______________________

**Basic information of the horse**

Name: ___________________________

Year of birth: ___________

Gender:

□ Mare
□ Gelding
□ Stallion

Breed:

□ Warmblood
□ Standardbred
□ Coldblood
□ Finnhorse
□ Pony
□ Other

If other breed, specify: _________________

Intended use:

□ Riding
□ Trotting
□ Working
□ Breeding
□ Other

If other intended use, specify: _______________________

Horse's health conditions: ______________________________________

Has your horse been previously identified as an MRSA/ESBL carrier?

□ No
□ Yes
□ I don't know

**Reason for the hospital visit**

Reason for the visit

□ Scheduled clinic appointment
□ Emergency visit (no appointment)

Briefly describe the reason for the visit (e.g., colic, lameness examination): _____________________

**Horse's home stable**

Postal code (or municipality if the postal code is unknown): _________

Stable type:

□ Private stable
□ Riding school
□ Other

If other stable type, specify: ______________________

Number of horses at the home stable (provide an estimate if the exact number is unknown): ______

Type of stall where your horse resides:

□ Individual stall
□ Open shed
□ Other

If other type of stall, specify: ________________________

Does the horse have the opportunity for muzzle contact with other horses?

□ Yes
□ No

Are there other animals in the stable environment? (Select all that apply):

□ Cat
□ Dog
□ Cattle
□ Pig
□ Other

If other animal, specify: __________________________

**Close contacts**

Has any horse in close contact with your horse been identified as an MRSA/ESBL carrier?

□ No
□ Yes
□ I don't know

Has any person in close contact with your horse been identified as an MRSA/ESBL carrier?

□ No
□ Yes
□ I don't know

Is the horse regularly handled by more than one person?

□ No
□ Yes
□ I don't know

Is the horse regularly handled by a person working in the healthcare field?

□ No
□ Yes
□ I don't know

The MRSA type common in horses is prevalent in pigs. Is the horse regularly handled by a person working on a pig farm?

□ No
□ Yes
□ I don't know

**Visits outside the home stable**

Has your horse attended an equine event in the last 3 months?

□ No
□ Yes
□ I don't know

Has your horse stayed abroad in the last 3 months?

□ No
□ Yes
□ I don't know

Has your horse visited an equine hospital or clinic in the last 3 months?

□ No
□ Yes
□ I don't know

If your horse has been in the hospital in the last 3 months, specify the duration of the hospital stay (e.g., 5 days): ____________

**Procedures and medication**

Has your horse undergone any surgical procedures in the last 3 months?

□ No
□ Yes
□ I don't know

If you answered yes, specify the nature of the procedure: ________________________

Has your horse been treated with antibiotics in the last 3 months?

□ No
□ Yes
□ I don't know

If you answered yes, specify the active substance or brand name: _________________

Has your horse been treated with other medications in the last 3 months?

□ No
□ Yes
□ I don't know

If you answered yes, specify the active substance or brand name: _________________
